# Could gender equality in parental leave harm off-springs' mental health? a registry study of the Swedish parental/child cohort of 1988/89

**DOI:** 10.1186/1475-9276-11-19

**Published:** 2012-03-30

**Authors:** Lisa Norström, Lene Lindberg, Anna Månsdotter

**Affiliations:** 1 Department of Public Health Sciences, Karolinska Institutet, SE-171 76 Stockholm, Sweden

**Keywords:** Parents, Gender equality, Children, Anxiety, Depression

## Abstract

**Introduction:**

Mental ill-health among children and young adults is a growing public health problem and research into causes involves consideration of family life and gender practice. This study aimed at exploring the association between parents' degree of gender equality in childcare and children's mental ill-health.

**Methods:**

The population consisted of Swedish parents and their firstborn child in 1988-1989 (N = 118 595 family units) and the statistical method was multiple logistic regression. Gender equality of childcare was indicated by the division of parental leave (1988-1990), and child mental ill-health was indicated by outpatient mental care (2001-2006) and drug prescription (2005-2008), for anxiety and depression.

**Results:**

The overall finding was that boys with gender traditional parents (mother dominance in childcare) have lower risk of depression measured by outpatient mental care than boys with gender-equal parents, while girls with gender traditional and gender untraditional parents (father dominance in childcare) have lower risk of anxiety measured by drug prescription than girls with gender-equal parents.

**Conclusions:**

This study suggests that unequal parenting regarding early childcare, whether traditional or untraditional, is more beneficial for offspring's mental health than equal parenting. However, further research is required to confirm our findings and to explore the pathways through which increased gender equality may influence child health.

## Introduction

Mental ill-health among children and young adults is a rapidly growing public health problem in Sweden and many parts of the world [1,2]. Among the eslished causes of depression and anxiety are factors associated with family life during grow up, such as parental neglect or overprotection, parents' mental ill-health and substance misuse, and loss of parent [3-6]. Further, since females generally suffer more from mental ill-health conditions than males [5,7,8], the search for explanations should consider the gender system. This refers to the spectrum of social structures leading boys and girls, men and women, to take on different attitudes, behaviors and works, and to an unequal division of power between the sexes [9]. Hence, a contributor to mental health may be experiences from growing up regarding gendered aspects of family life, in other words, from how the parents positioned themselves in terms of gender equality.

There are two main pathways available: 1) the degree of gender equality between parents affects the child's gendered views and practices, which in turn affect their mental health via mechanisms linked to the gender system during childhood, adolescence, and adulthood; 2) the degree of gender equality between the parents affects the child's mental health via parental mechanisms such as agreement or disagreement regarding divisions of duties, multiple role conflicts or benefits, decisions on living together or separated, and belonging to a pioneer or laggard family in terms of gender equality, and via impact on the mothers' and fathers' health and well-being.

### Gender and mental ill-health

The first pathway of parents' gender equality affecting children's gendered views and practices, and hence their mental health prospects, is based on the idea that the gender system, or in short gender, affects plentiful aspects of life [9]. However, recent research indicates only partial support for the inheritance of childhood gender experiences to own gendered life; it seems to be more valid for women than for men [10].

Differences regarding access to resources, methods of coping with stress, styles of interacting with others, self-images, expectations of others, etc. are all factors that may influence mental health positively or negatively [7,9]. First, the unequal levels of power, influence, and resources are likely to create feelings of subordination and decreased mental health among females as compared to males in all stages of life [11]. Further, embracing traditional femininity traits such as affection, warmth, tenderness, gentleness, sensitivity to others' needs, eagerness to soothe hurt feelings, and caring for children may lead to worries and despair, and hence increased risk of mental ill-health among women. However, these traits may also protect mental health [12]. Concurrently, embracing traditional masculinity traits such as independence, dominance, strength, defending own beliefs, and willing to take risks may both risk and protect from mental health problems among men [13,14].

An early proposal was that that performing well on masculinity is healthy for men, while performing well on femininity is healthy for women [15]. That is, discrepancies between the gender ideals adopted from childhood and the surrounding, and actual gender practices, may risk confusion and conflict, and hence poor mental health for both sexes [16]. This idea that acting according to gender ideals is necessarily beneficial, was later challenged by the proposal that androgyny is the best position in terms of health [12]. That is, both sexes were assumed to be able of, and to gain from, embracing both femininity and masculinity ideals [17].

Naturally, mental ill-health includes a number of conditions and diagnoses, which further complicate the picture [8]; for example, femininity appears to be protective against antisocial behavior and substance abuse, while masculinity appears to be protective against feeling low and nervous [11]. The conclusion from a review by Piccinelli and Wilkinson [5] was that the following factors contribute to the gender gap in depression: females are at greater risk of sexual abuse in childhood; females are at greater risk of anxiety and depression at earlier stages of life; females suffer more from role limitation associated with lack of choice, role overload, and competing roles; females do not experience more adverse life events, but suffer more from them due to social conditions.

### Parents' gender equality and mental health

The second pathway of gender equality affecting aspects of the parents' life, and hence the children's mental health prospects, is based on the idea that challenging gender norms may imply beneficial as well as detrimental consequences. A well-established theory is that children's mental health is likely to improve when their parents become more gender-equal in the caring and breadwinning roles of parenthood [18,19]. However, the empirical evidence on how gender equality in terms of "more or less similarity between the mother and father" [20], affects child health is lacking. The proposal of parental equality being beneficial for children is therefore likely to be based on studies examining effects from, for example, having a "working mother", and an "involved father".

It has been found that the emotional tone was more positive among fathers who were primary caregivers than other fathers, and that children of primary care giving fathers were happier during play compared to other children [21]. Other studies have reported that the involvement of fathers during childhood is crucial for children's emotional development [22], and protective against mental distress during adolescence [23]. In a study by Pruett [24] was shown that fathers' childcare, measured by paternity leave, associates with capacity to solve problems and social skills among infants. The overall conclusion from a review of longitudinal studies was that father involvement predicts a range of positive outcomes among children. Particularly, it may prevent risky lifestyles among boys and mental problems among girls [25]. These results suggest that fathers' caring is a determinant for children's mental health, independently, however, of the mother's caring.

Research on how gender equality affects children's mental health is generally assuming that mothers and fathers are quite similar in doing parenting [26]. Nevertheless, women usually take on the breadwinning role of parenthood before men take on the caring role, which have placed women at risk of stressful demands and double workload [27]. This means that gender equality of childcare should decrease women's stress from multiple roles [28]. Since men tend to have fewer roles, they are also likely to benefit from gender equality in childcare according to the theory of expansion [29]. That is, from being able to tackle difficulties in one sphere (e.g. work) with positive circumstances in other spheres (e.g. family). In register-based studies among Swedish parents of 1978, the proposal that both sexes would gain health from gender equality in childcare has been supported. Gender-equal fathers in terms of taking temporary childcare days ran decreased risk of later alcohol-related problems, sickness absence and premature death, and gender-equal women ran decreased risk of sickness absence [30,31]. Parental sickness and loss are stressful events for children, who therefore are likely to benefit from parental equality in terms of mental health.

Parents' progress towards gender equality may also involve adverse mental health effects for their children. One may expect increased negotiations, and perhaps quarrels, among couples where only one parent wants to change the gender traditional division of everyday work, from which the child may be harmed [32]. Current evidence indicates that fathers involved in childcare experience negative pressure from individuals in the surrounding [33], but also that paternity leave decreases the risk of divorce and separation [34]. Mental health prospects may additionally be affected by the interaction between the parents' and the surrounding's practices regarding gender equality. However, since fathers tend to benefit whilst women tend to detriment in sickness from being a "gender pioneer" [35], the net effect on children's mental health is hard to predict. And even though both parents gain health and well-being from altering duties [33], the child could experience mental stress from feeling different in a society where other families practise gender traditional norms [16]. Finally, a couple's ambition of gender equality in childcare could be linked to a career ambition among both parents, which may harm the child by reduced overall parental engagement, interaction, and support [33].

### Objective

The objective of the present study was to explore gender equality in childcare - by means of the parents' division of parental leave, in relation to mental ill-health among children - by means of outpatient mental care for anxiety and depression, and prescription of anxiety drugs and anti-depressants. The reason for selecting parental leave is that it represents an early indicator of caring duties, and that it is assumed to have long-lasting effects on the division of family/caring and work/breadwinning life [18]. The reason for choosing outpatient mental care as well as drug prescription outcomes is that this broadens the indication (note, not occurrence) of mental ill-health [8]. Since theories on parental equality and child mental health are opposing and empirical results are lacking, our hypotheses were restricted to: 1) the degree of gender equality between parents may affect mental conditions among children, either beneficially or detrimentally, 2) the associations may vary between boys and girls since the gender system imposes gender-stratified experiences from early childhood.

## Methods

### Population

The population was based on a cohort of all mothers and fathers in Sweden who had their first child together in 1988 and 1989, along with these children (N = 118 595 family units). If a parent died during the period of measuring parental leave, or during follow-up, or if none of the parents used the parental leave entitlement for at least one day, the family unit was excluded. This resulted in a study population of 105 786 family units in the crude model, 54 282 boys and 51 504 girls. Due to missing data in the confounding variables, the final model consisted of 99 113 family units; 50 859 boys and 48 524 girls.

### Exposure: gender equality of parental leave

The data on parental leave, for both mothers and fathers, come from the Swedish Social Insurance Register (Social Insurance Agency) and consist of days with full payment during the period 1988-1990. The parental leave days offered per child were 360 for those having a child during January 1988-June 1989, and 450 for those having a child from July 1989, due to a legislative change. For the first period, 270 days were paid by 90% of income, for the second period, 360 days were paid by 90% of income, and for both periods, additionally 90 days were paid at a guaranteed level of 60 Swedish crowns (SEK) per day (equivalent to 8.32 USD). The inclusion of family units of both periods is relevant since both parents were exposed to the same offer of parental leave, and since the study considers their relative position. The right to parental leave in Sweden requires that the parent (or other person with legal custody) is registered at the Social Insurance Agency. That is, it is not linked to employment per see (a housewife may receive it, a part-time employee may extend the period of leave by taking part-time leave, etc.), but to income (the housewife with no income received the guaranteed level of 60 SEK per day, the part-time employee reduced his/her income and hence the level of payment, etc.)

The division of days on parental leave between the mother and the father was selected to indicate the parental couple as gender-equal or gender-unequal in childcare. The unequal couples were further divided based on the traditional gender norm of female dominance in caring (and male in breadwinning), and the untraditional gender norm of male dominance in caring (and female in breadwinning).

Based on the guiding principle in Swedish gender policies [18], and on the theoretical notion that a gender-equal society requires that both sexes participate more or less equally in every sphere of life including childcare [20], equality was defined when each parent took at least 40% and at most 60% of the total parental leave. Based on earlier empirical studies [30,31], inequality was further distinguished by the limit of one parent taking less than 20% (and hence the other parent more than 80%) of the parental leave days. This resulted in five categories: very traditionally unequal couples (mother more than 80% of the parents' total of parental leave days); rather traditionally unequal (mother more than 60% and less than 80%t); equal (both parents between 40-60%); rather untraditionally unequal (father more than 60% and less than 80%); very untraditionally unequal (father more than 80%). In the regression model, however, the categories of very and rather untraditional were collapsed into one category (untraditional) due to few family units.

### Outcomes: indicators of mental ill-health

The outcomes of mental ill-health were measured by at least one visit to outpatient mental care for depression or anxiety, and at least one prescription of anti-depressants or anxiety drugs. The information was obtained from the Outpatient Register and the Drug Register (National Board of Health and Welfare). The International Classification of Diseases (ICD-10) codes used regarding outpatient mental care were for depression: F32.0-F39.9, and for anxiety: F40.0-F45.9 F48.0-F48.9 F93.0-F93.0. The Anatomic Therapeutic Chemical classification system (ATC) codes used were for anti-depressants: N06A, and for anxiety drugs: N05B and N05C. The follow-up periods were 2001-2006 for outpatient mental care (i.e. age of child 13-18 years), and 1 July 2005-31 July 2008 for drugs (i.e. age of child 17-20 years). The reason for using different periods, and hence, ages of the child, is solely technical; regarding the starting date, the Outpatient register reached national coverage by January 2001, while the Drug register reached national coverage by July 2008; regarding the ending date, outpatient care diagnoses were reported with larger time lags than drug prescriptions to the national registers, respectively.

The relevant correlations between the four outcomes according to Pearson coefficient (1 representing perfect positive correlation, -1 representing perfect negative correlation) were: between outpatient mental care for depression and outpatient mental care for anxiety 0.265, between outpatient mental care for depression and anti-depressants 0.322, and between outpatient mental care for anxiety and anxiety drugs 0.182 (all statistically significant at the 5% level).

### Confounders

There are a number of factors that may confound the association between gender (in)equality based on the categorization of parental leave and the considered mental ill-health outcomes among children. A basic consideration is that gender-equal parents took fewer days of parental leave than traditional parents and more days than untraditional parents, which indicates different caring ambitions and possibly different mental health prospects among children. Since this study seeks to examine potential impact from the relative position of parental leave, the parents' total number of days on leave (1988-1990) was controlled for.

The demographic and socioeconomic confounders examined and controlled for regarding each parent were: having another child 1990, year of birth, born outside Sweden, married or living together 1990, educational level 1990 (9 years of compulsory schooling, 11-12 years of secondary schooling, tertiary schooling), and income 1987 (from employment). The possibility that the parents' mental ill-health and abuse (which is likely to affect the child's mental ill-health) differ by gender equality category was controlled for by institutional care of parents 1986-90 due to psychosis, depression, anxiety, and alcohol-related diagnoses.

### Statistical analyses

The statistical method used was multiple logistic regressions, by sex and in total. The odds ratios (OR) were assumed to estimate the relative risks (RR) due the relatively low prevalence of the outcomes (from 1.2% regarding outpatient mental care for depression to 5% regarding anxiety drugs). The reference group was children of parents who shared parental leave equally (within the range 40-60%), i.e. the results demonstrate the increased/decreased risk of mental ill-health among children with very traditional, rather traditional, and untraditional parents compared to children with equal parents. The reason was that earlier studies have indicated that the association between the exposure categories (from traditional, via equal, to untraditional) and health outcomes is curvilinear [30,31], and that interpretation of results generally gains from looking at deviations from gender equality. The confounders were added in groups to the crude analysis; first step: controls for the parents' total amount of parental leave, year of birth, country of birth, married/living together, and having another child; second step: additional controls for the parents' education and income; and third step: additional controls for the parents' institutional care history (the crude and fully adjusted results are shown in tables).

The analyses were performed in SPSS Statistics version 17.0, and statistical significance was reported as *p*-values or confidence intervals (CI) at the 5% level. The study has been approved from an ethical point of view by the Stockholm Board of Ethical Clearance (No: 2008/363-31/5).

## Results

The distribution of gender (in)equality categories by mental ill-health outcomes, and of confounders by gender (in)equality categories are reported in Tables 1, 2. The results confirm the picture of differences in mental ill-health by sex, since girls are generally twice as affected as boys in all outcomes. It is also shown that prescription of drugs (3.5 years of follow up) is more common than visits to outpatient mental care (6 years of follow up) regarding both depression and anxiety, for both sexes. At the descriptive level, the mental ill-health outcomes do not vary by (in)equality category, whilst the confounders (except for fathers and institutional care for psychosis, depression, and for mothers and institutional care for alcohol use) vary by (in)equality category.

**Table 1:** Distribution of parental couples, and of outpatient mental care for anxiety and depression (2001-01-01-2006-12-31), and prescription of anxiety drugs and anti- depressants (2005-07-01-2008-07-31) among children, between categories of gender (in)equality; by sex and in total; p-value chi-square test

Mental ill-health: Gender (in)equality:	Distribution of parental couples	Outpatient mental care Anxiety	Outpatient mental care Depression	Anxiety drugs	Anti-depressants
***Boys:***					
Very traditional	47 506 (87.5%)	0.9%	0.7%	3.6%	3.1%
Rather traditional	4 483 (8.3%)	0.9%	0.7%	3.5%	3.1%
Very untraditional	1 317 (2.4%)	1.3%	1.1%	3.6%	3.3%
Equal	420 (0.8%)	1.4%	1.4%	4.3%	2.9%
Rather untraditional	556 (1.0%)	0.7%	1.1%	3.4%	2.3%
Total	54 282	516 (1.0%)	370 (0.7%)	1 940 (3.6%)	1 682 (3.1%)
*p-value chi-square test distributions*		*0.536*	*0.052*	*0.948*	*0.840*

***Girls:***					
Very traditional	45 106 (87.6%)	2.2%	1.8%	6.6%	6.7%
Rather traditional	4 212 (8.2%)	2.2%	1.9%	6.4%	7.0%
Equal	1 234 (2.4%)	1.5%	2.1%	8.7%	7.7%
Rather untraditional	415 (0.8%)	1.9%	1.0%	5.1%	5.8%
Very untraditional	537 (1.0%)	2.4%	2.2%	6.1%	7.3%
Total	51 504	1 106 (2.1%)	920 (1.8%)	3 402 (6.6%)	3 462 (6.7%*)*
*p-value chi-square test distributions*		*0.529*	*0.506*	*0.031*	*0.504*

***Both:***					
Very traditional	92 612 (87.5%)	1.5%	1.2%	5.0%	4.8%
Rather traditional	8 695 (8.2%)	1.5%	1.3%	4.9%	5.0%
Equal	2 551 (2.4%)	1.4%	1.6%	6.0%	5.4%
Rather untraditional	835 (0.8%)	1.7%	1.2%	4.7%	4.3%
Very untraditional	1 093 (1.0%)	1.6%	1.6%	4.8%	4.8%
Total	105 786	1 622 (1.6%)	1 290 (1.2%)	5 342 (5.0%)	5 144 (4.9%)
*p-value chi-square test distributions*		*0.967*	*0.235*	*0.200*	*0.599*

**Table 2:** Confounders by category of gender (in)equality; means or proportions, for both parents, or for fathers (F) and mothers (M), respectively; 95% CI around means *p*-value chi-square test for proportions

Gender (in)equality category: Confounder:	Very Traditional	Rather Traditional	Equal	Rather Untraditional	Very Untraditional	*p-value chi-square test*
Total parental leave	Both: 412	Both: 395	Both: 392	Both: 369	Both: 267	
1989-90 (mean days)	(411-413)	(392-398)	(387-397)	(360-378)	(258-276)	
Second child 1990 (%)	F: 18.1	F: 17.8	F: 12.6	F: 13.7	F: 8.8	*0.000*
	M: 18.4	M: 18.1	M: 12.9	M: 13.4	M: 9.0	*0.000*
Year of birth	F: 1959	F: 1959	F: 1958	F: 1958	F: 1956	
	M: 1962	M: 1961	M: 1961	M: 1961	M: 1961	
Married/living together 1990 (%)	F: 86.7	F: 93.5	F: 93.2	F: 89.5	F: 89.1	*0.000*
	M: 86.2	M: 93.5	M: 93.2	M: 89.3	M: 90.1	*0.000*
Born outside Sweden (%)	F: 13.0	F: 9.5	F: 12.7	F: 20.2	F: 27.9	*0.000*
	M: 11.5	M: 10.6	M:14.1	M: 25.6	M:43.9	*0.000*
Income from employment (mean SEK)	F: 102 010 (102 006-102 014)	F: 106 742 (106 731-106 753)	F: 101 801 (101 779-101 823)	F: 94 238 (94 200-94 276)	F: 94 246 (94 226-94 266)	
	M: 74 211 (74 208-74 214)	M: 78 381 (78 371-78 391)	M:68 514 (68 414-68 534)	M: 49 059 (49 024-49 094)	M: 29 789 (29 762-29 816)	
Education, highest (%)	F: 21.8	F: 34.1	F: 35.9	F: 29.0	F: 34.4	*0.000*
	M: 21.9	M: 39.2	M: 40.3	M: 34.4	M:32.2	*0.000*
Institutional care 1986-89:						
Psychosis (%)	F: 0.1	F: 0.1	F: 0.1	F: 0.2	F: 0.4	*0.071*
	M: 0.2	M:0.3	M: 0.4	M: 0.6	M: 1.6	*0.000*
Depression (%)	F: 0.2	F: 0.1	F: 0.2	F: 0.1	F: 0.1	*0.735*
	M: 0.3	M: 0.4	M: 0.5	M: 0.6	M: 1.6	*0.000*
Anxiety (%)	F: 0.5	F: 0.3	F: 0.2	F: 0.5	F: 0.7	*0.052*
	M: 0.7	M: 0.8	M: 1.0	M: 1.3	M: 2.5	*0.000*
Alcohol abuse (%)	F: 0.7	F: 0.3	F: 0.3	F: 0.6	F: 0.8	*0.000*
	M:0.2	M:0.2	M: 0.2	M: 0.2	M: 0.2	*0.914*
Separation 1991-2006 (%)	Both: 47.9	Both: 41.6	Both: 45.6	Both: 49.6	Both: 50.5	*0.000*

The results from the regression analyses are reported in Table 3 (outpatient mental care) and Table 4 (drug prescription). An overall observation is that adjusting for confounding variables has a relatively small impact on the association between gender equality in childcare during early parenthood and future risk of mental ill-health of the children.

**Table 3:** Odds ratios of outpatient mental care for anxiety and depression (2001-01-01-2006-12-31) of the child by category of gender (in)equality of the parents; crude and fully adjusted results, by sex and in total; 95% CI

	Odds ratios Crude Boys *n:54 282*	Odds ratios Crude Girls *n:51 504*	Odds ratios Crude Both *N:105 786*	Odds ratios Adjusted* Boys *n:50 859*	Odds ratios Adjusted* Girls *n:48 254*	Odds ratios Adjusted* Both *N:99 113*
**Anxiety outpatient mental care**						
Very traditional	0.73	1.49	1.12	0.69	1.48	1.07
	(0.45-1.19)	(0.93-2.38)	(0.80-1.57)	(0.42-1.13)	(0.90-2.45)	(0.75-1.52)
Rather traditional	0.69	1.52	1.12	0.67	1.66	1.14
	(0.39-1.22)	(0.92-2.54)	(0.77-1.62)	(0.37-1.19)	(0.97-2.83)	(0.78-1.68)
Equal	1	1	1	1	1	1
Untraditional	0.79	1.52	1.18	0.62	1.27	0.95
	(0.36-1.74)	(0.81-2.88)	(0.72-1.91)	(0.25-1.50)	(0.61-2.63)	(0.55-1.65)

**Depression outpatient mental care**						
Very traditional	0.57	0.84	0.74	0.57	0.83	0.74
	(0.34-0.96)	(0.56-1.24)	(0.54-1.02)	(0.33-0.98)	(0.55-1.25)	(0.53-1.03)
Rather traditional	0.62	0.90	0.80	0.67	0.94	0.84
	(0.34-1.16)	(0.58-1.41)	(0.56-1.15)	(0.35-1.26)	(0.59-1.50)	(0.58-1.23)
Equal	1	1	1	1	1	1
Untraditional	1.08	0.79	0.90	0.98	0.83	0.89
	(0.50-2.32)	(0.42-1.49)	(0.56-1.46)	(0.42-2.29)	(0.43-1.63)	(0.53-1.51)

*) Adjusted for: parents' total amount of parental leave, age, country of birth, living together or not, getting a second child during exposure period, parents' income and educational level, and parents' institutional care for psychosis, anxiety, depression, and alcohol abuse

**Table 4:** Odds ratios of prescription of anxiety drugs and anti- depressants (2005-07-01-2008-07-31) of the child by category of gender (in)equality of the parents; crude and fully adjusted results, by sex and in total; 95% CI

	Odds ratios Crude Boys *n:54 282*	Odds ratios Crude Girls *n: 51 504*	Odds ratios Crude Both *N:105 786*	Odds ratios Adjusted* Boys *n:50 859*	Odds ratios Adjusted* Girls *n:48 524*	Odds ratios Adjusted* Both *N:99 113*
**Anxiety drugs**						
Very traditional	1.00	0.74	0.83	0.97	0.72	0.80
	(0.75-1.35)	(0.61-0.91)	(0.70-0.98)	(0.71-1.32)	(0.58-0.89)	(0.67-0.95)
Rather traditional	0.98	0.72	0.80	1.01	0.77	0.85
	(0.70-1.37)	(0.57-0.91)	(0.66-0.97)	(0.72-1.43)	(0.61-0.98)	(0.70-1.03)
Equal	1	1	1	1	1	1
Untraditional	1.06	0.63	0.77	0.82	0.58	0.66
	(0.69-1.65)	(0.45-0.89)	(0.59-1.01)	(0.49-1.35)	(0.40-0.85)	(0.49-0.89)

**Anti-depressants**						
Very traditional	0.93	0.86	0.88	0.89	0.85	0.87
	(0.68-1.26)	(0.69-1.06)	(0.74-1.05)	(0.65-1.22)	(0.68-1.06)	(0.73-1.04)
Rather traditional	0.92	0.90	0.91	0.93	0.95	0.94
	(0.65-1.30)	(0.71-1.14)	(0.74-1.10)	(0.65-1.32)	(0.74-1.22)	(0.77-1.16)
Equal	1	1	1	1	1	1
Untraditional	0.76	0.85	0.83	0.59	0.85	0.78
	(0.46-1.25)	(0.61-1.18)	(0.63-1.09)	(0.33-1.05)	(0.60-1.21)	(0.58-1.05)

*) Adjusted for: parents' total amount of parental leave, age, country of birth, living together or not, getting a second child during exposure period, parents' income and educational level, and parents' institutional care for psychosis, anxiety, depression, and alcohol abuse

Regarding outpatient mental care for anxiety, none of the results are statistically significant, neither crude nor after adjusting for confounders. Regarding outpatient care for depression, boys with very traditional parents are shown to have a 43% lower risk than boys with gender-equal parents, in the crude as well as adjusted model.

Regarding anxiety drugs, it is shown that girls with very traditional, rather traditional and untraditional parents have lower risks than girls with gender-equal parents. The decreased relative risks remain after adjusting for confounders: 28% if very traditional, 23% if rather traditional, and 42% if untraditional. Regarding anti-depressants, none of the crude or adjusted results are found to be statistically significant.

A set of sensitivity analyses, such as excluding parents who had been in institutional care due to psychosis, depression, anxiety, and alcohol-related diagnoses instead of controlling for this information, and altering the reference group from equal parents to very traditional parents, did not essentially change the results. That is, the risk estimates reported seem to be robust.

## Discussion

### Main findings

This study has confirmed that girls consume around twice as much outpatient mental care in the ages 13-18 years, and drugs due to anxiety and depression in the ages 17-20 years, than boys. This supports the relevance of exploring aspects of the gender system in relation to mental ill-health, here in terms of potential effects on children from the mother's and father's division of childcare duties indicated by parental leave. The main findings were that boys with gender traditional parents run lower risk of depression measured by outpatient mental care, while girls with gender traditional *and *gender untraditional parents run lower risk of anxiety measured by drug prescription, compared to those with gender-equal parents.

### Earlier research

The pathways assumed regarding the link between parents' equality and children's mental ill-health were: first, the degree of gender equality between parents affects gendered views and practices among children, which in turn affects their mental health; second, the degree of gender equality between parents affects children's mental health via various parental mechanisms. The present study was not designed for examining mediating mechanisms, neither among the children nor the parents. The reason for focusing on the second pathway below is that the extent to which individuals bring their childhood gendered experiences is hesitant [10].

Our understanding from earlier research is that gender equality between parents is good for the children. The overall conclusion from a review was, for example, that having an engaged father benefits both psychological and physical aspects of health among children of both sexes [25]. It has also been found that generous parental leave policies associate with physical aspects of health such as increased birth weight, decreased premature birth, and decreased mortality among infants at the ecological level [36], that dual-earner regimes offering parental leave to both parents have lower levels of child poverty and child mortality [37], and that both maternity leave [38] and paternity leave [39] have positive effects on length of breastfeeding, and child mortality.

The central issue of debate is hence why we found that gender equality is negative for children in terms of mental health. That is, why are girls with gender traditional parents more protected against anxiety (measured by drugs) than girls with equal parents, and why boys are with gender traditional parents more protected against depression (measured by outpatient care) than boys with equal parents?

First to note is that earlier studies do not explicitly examine how the relative position of parents regarding childcare (parental leave) associates with children's mental health. Nevertheless, our assumption that gender equality of childcare would be good for mothers according to the theory of released stress [28], for the father according to the theory of expansion [29], and hence for the child because of the increased health of her/his parents, was rejected. It may also indicate that the decreased risk of adverse health outcomes among gender-equal parents in temporary childcare, found in earlier studies of Swedish parents [30,31], not evidently transform into mental health benefits for the child.

Instead, our results support that increased gender equality harms children by, for example, increased uncertainty and conflict between parents in everyday life, which should be especially evident if one parent dominates the decision [32]. It could also be that the overall effect from being a pioneering family in terms of gender equality is detrimental for the parents' well-being, and hence for the child's mental health prospects [35]. Further, even though the parents are comfortable with sharing childcare duties equally, and to reject gender stereotypes, their child could still experience mental pressure from being different from children in gender traditional families [33]. The results may also include the possibility that both parents in the gender-equal category have a relative career versus caring ambition. That is, gender equality in the present study could represent absence of long-term caring, and therefore, lower parental engagement, interaction, and support [33].

The results regarding traditional versus equal parents support the view that mothers should take care of children, and hence, the argument of a continued division of female caring and male breadwinning [20]. Yet, our study adds to this theoretical (ideological) framework a potential protection from anxiety measured by drug prescription among girls with untraditionally unequal parents as well. This suggests that the one-parent caring is beneficial rather than the mother-only caring, which is supported by research showing that gender does not affect the ability to take care of and respond to children's signals and needs [40]. However, being untraditional in childcare by means of fathers taking most parental leave is indeed a challenge against the gendered norm of family life, and should also include uncertainty and conflict between parents, difficulties from pioneering in gender equality, etc. Whether the benefits from consistency of care may dominate over the risks discussed for equal parents is an example of what we believe should be scrutinized in future studies.

A final interpretation, based on the measures used for indicating mental ill-health, is that equal (and untraditional) parents may be particularly observant about their children's problems leading them to greater medical attendance. That is, it could be the result of selection bias where a certain type of "conscious parenthood" involves ambitions of gender equality as well as ambitions of healthy children [41]. Based on ancient views regarding gender and health, a tentative proposal is that even gender-equal parents and health care professionals could be more prone to direct girls to anxious (hysteric), and boys to depressive (melancholic) signs and diagnoses [20].

### Strengths and weaknesses

The main strength of the present study is the large study population encompassing all Swedish couples who had their first child together in 1988-1989, together with reliable registry data on parental leave for both parents, four indicators of mental ill-health regarding the children, and several confounding factors. Obviously, there are a number of weaknesses as well.

One may call in question the division of parental leave as an indicator of the division of childcare between parents, since it does neither consider caring duties during evenings and weekends, nor the quality of the caring performed. One must also consider that the outcomes of mental ill-health involve propensity to seek care, which may vary from actual mental ill-health, and that the measures involves a time lag, due to outpatient mental care and drug prescription just recently being covered in Swedish national registers.

Another weakness is that the study presents associations between parents' gender equality and children's mental ill-health, and speculates about pathways, but does not offer any empirical explanations. Parents defined as gender-equal may have been unique in aspects not controlled for. That is, the risk of mental ill-health among their children could be caused by lack of caring capacity, intense career ambition, proneness to consume care, etc. rather than by their division of parental leave. However, parental leave is assumed to represent a valid indicator of childcare ambition and practice among individuals, and a determinant for gender equality in general [18].

Finally, the parental cohort is from the late 1980s. This means that the consequences among parents and children from being different from the gendered norm regarding family life could have been greater in the studied population than among gender-equal families of today. Sweden was the first country in the world that permitted fathers to take paid parental leave in 1974, with the motive of enhancing the potential for males in the sphere of caring, and for females in the sphere of working [42]. Since then, fathers' share of parental leave has developed greatly, from 0.5% in 1974, to 5.2% in 1980, 7.4% in 1990, 12.4% in 2000, and 23.1% in 2010 (Social Insurance Agency). One explanation to the increase is the introduction of the so called "daddy-days", i.e. the non-transferable reservation of 30 days to one parent by January 1995, and the extension to 60 days by January 2002. As a whole, the transformation of our findings to the current Swedish situation should be done with caution. The relevance for other countries is dependent on the degree of formal and informal opportunities to act against traditional gender norms regarding family life in the specific setting.

## Conclusions

Progress towards a gender-equal parenthood could have beneficial as well as detrimental effects on the mental health status of children. This study suggests that having gender-equal parents regarding early childcare (parental leave) implicates higher risk of outpatient mental care for depression among boys (compared to having traditional parents), and higher risk of drug prescription for anxiety among girls (compared to having traditional and untraditional parents). There are several potential explanations to our results, and we recommend that the study should be considered tentatively while waiting for support or contradiction in future research.

## Competing interests

The authors declare that they have no competing interests.

## Authors' contributions

LN performed the statistical analyses in collaboration with AM, and wrote the first draft of the paper. LL was responsible for aspects regarding the mental ill-health outcomes. AM was responsible for the original construction of the cohort, the collection of data and the theoretical aspects of gender equality. All authors contributed to the intellectual work and choice of methods, and contributed to the writing of the present manuscript version.
